# Identification and characterization of the structure–activity relationships involved in UGT1A1 inhibition by anthraquinone and dianthrone constituents of *Polygonum multiflorum*

**DOI:** 10.1038/s41598-017-18231-y

**Published:** 2017-12-20

**Authors:** Qi Wang, Yadan Wang, Yong Li, Binyu Wen, Zhong Dai, Shuangcheng Ma, Yujie Zhang

**Affiliations:** 1 Beijing University of Chinese Medicine, Beijing, 100029 China; 2 National Institutes for Food and Drug Control, Beijing, 100050 China; 3 Dongfang Hospital, Beijing University of Chinese Medicine, Beijing, 100078 China

## Abstract

The adverse effects of *Polygonum* (*P*.) *multiflorum*, including abnormal bilirubin metabolism, are a serious public health issue. As uridine diphosphate (UDP)-glucuronosyltransferase 1A1 (UGT1A1) is the only enzyme responsible for bilirubin metabolism, we investigated the inhibitory effect of a *P*. *multiflorum* extract and 10 anthraquinone and dianthrone compounds on UGT1A1 in rat liver microsomes *in vitro*. The *P*. *multiflorum* extract exhibited the strongest inhibitory effect on UGT1A1 activity (inhibition constant [K_i_] = 0.3257 μM, 1422 μg of material/mL), followed by cis-emodin dianthrones (K_i_ = 0.8630 μM), trans-emodin dianthrones (K_i_ = 1.083 μM), emodin-8-O-glc (K_i_ = 3.425 μM), and polygonumnolide C2 (K_i_ = 4.291 μM). Analysis of the structure–activity relationships of these compounds suggested that the spatial orientation of the molecules and the presence of particular functional groups affect UGT1A1 inhibition. A mechanistic analysis showed that all the tested compounds docked into two of the nine active sites of UGT1A1 and suggested that hydrophobic interactions and hydrogen bonds are important for the affinity of the tested compounds for UGT1A1; moreover, their interaction energies were generally in agreement with the K_i_ values. These findings provide insight into adverse reactions to *P*. *multiflorum* and identify the pharmacophores involved in inhibition of UGT1A1.

## Introduction

*P*. *multiflorum* (common names: *Polygonum multiflorum* Thunb^1^, Polygoni Multiflora Radix^2^, *Fallopia multiflora*, Heshouwu^3^, fleeceflower root^4,5^, Polygoni multiflori^6^, and foti root^7^) is among the most frequently used medicinal plants in China. The main components of this plant are flavonoids^1,8–11^, quinones, and anthrones^12^, which are used as ingredients in various medicines. The major quinone constituents of *P*. *multiflorum* have multiple biological activities, exerting antimicrobial, antioxidant, and anti-human cytomegalovirus effects^13^. However, *P*. *multiflorum* reportedly also exerts adverse effects^14–18^, the most common and serious of which is abnormal bilirubin metabolism^19–21^. Also, anthraquinones can lead to elevated total bilirubin levels and induce hepatotoxicity^22–30^.

Uridine diphosphate (UDP)-glucuronosyltransferases (UGTs) are an important family of phase II drug-metabolizing enzymes that play major roles in the elimination and detoxification of endogenous and exogenous compounds^31–33^. UGT1A1 is the only UGT isoform involved in the metabolic clearance of bilirubin, which is a toxic waste product of heme degradation^34^. The inhibition of UGT1A1 may therefore lead to bilirubin accumulation, which can induce, for example, jaundice, liver dysfunction, and carcinogenesis^35–38^.

Therefore, we hypothesized that the abnormal bilirubin metabolism and hyperbilirubinemia observed following administration of *P*. *multiflorum* could be a result of UGT1A1 inhibition, primarily by quinones.

We tested this hypothesis previously by administering a 70% ethanol extract of *P*. *multiflorum* orally to rats, which resulted in marked inhibition of UGT1A1 activity^39^. Furthermore, we recently demonstrated that emodin competitively inhibits UGT1A1 in three model systems (K_i_ = 5.400 ± 0.956 (p < 0.05) in the HLM system, 10.02 ± 0.611 (p < 0.05) in the RLM system, and 4.850 ± 0.528 (p < 0.05) in the rUGT1A1 system). The degree of inhibition of rat and human UGT1A1 did not differ significantly^40^.

**Figure 1:**
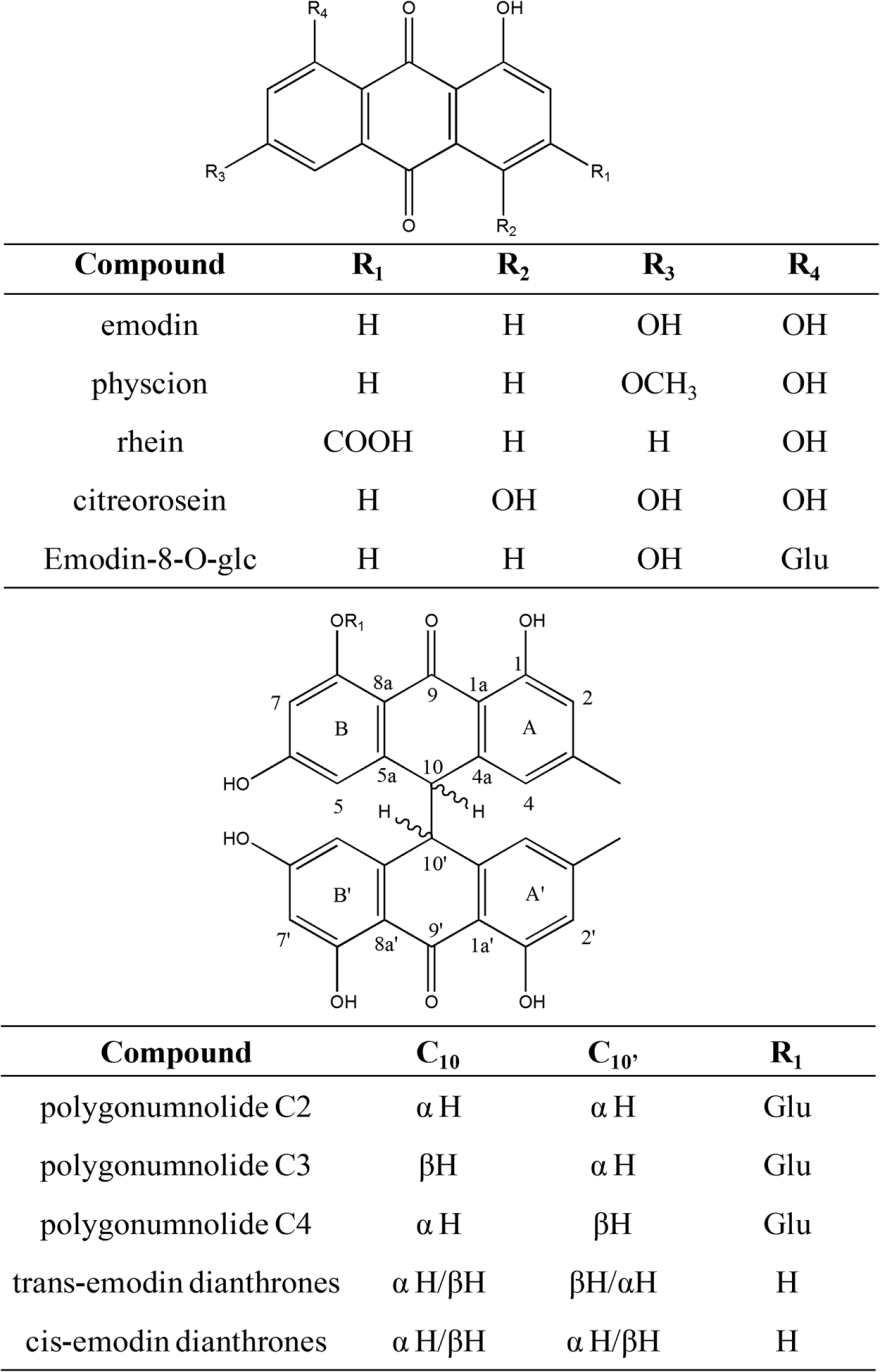
Chemical structures of 10 *P*. *multiflorum* compounds.

In the present study, we used a sensitive and robust *in vitro* assay to investigate UGT1A1 inhibition by a *P*. *multiflorum* extract and 10 individual components in a rat liver microsomes (RLM) system^41^. The monomer components tested were emodin-type anthraquinones and dianthrone derivatives (Fig. 1). Based on the results, we discuss the relationships between the structures of these compounds and their inhibitory effects on UGT1A1. Moreover, a mechanistic analysis was performed by the molecular docking method. Our findings will facilitate further studies of the mechanisms underlying the toxicity of *P*. *multiflorum*.

## Results

### Inhibitory effects of *P. multiflorum* extract and its constituents on UGT1A1

Different concentrations of an ethanol *P*. *multiflorum* extract and its 10 major components were screened for their inhibition of UGT1A1 activity in RLM. The second plots of the slopes of the Lineweaver–Burk plots were used to calculate K_i_ values. The modes of inhibition were competitive, non-competitive, mixed-competitive, and un-competitive (Table 1 and Supplementary Fig. S1). *P*. *multiflorum* extract (K_i_ = 0.3257 μM, 1422 μg of material/mL) exhibited the strongest inhibition of UGT1A1. The rank order of UGT1A1 inhibitory potency of the 10 tested major compounds was cis-emodin dianthrones (K_i_ = 0.8630 μM) > trans-emodin dianthrones (K_i_ = 1.083 μM) > emodin-8-O-glc (K_i_ = 3.425 μM) > polygonumnolide C2 (K_i_ = 4.291 μM) > emodin (K_i_ = 10.01 μM) > polygonumnolide C3 (K_i_ = 12.89 μM) > citreorosein (K_i_ = 18.56 μM) > polygonumnolide C4 (K_i_ = 77.42 μM) > physcion (K_i_ = 94.75 μM) > rhein (K_i_ = 127.3 μM).

**Table 1:** Modes of inhibition and K_i_ values for inhibition of UGT1A1-mediated metabolism by *P*. *multiflorum* extracts and 11 of its components in rat liver microsomes.

No.	Drugs	Mode of inhibition	*K*_*i*_ value (μM)
a	Polygonum multiflorum extracts	Competitive inhibition	0.3257
b	Emodin	Competitive inhibition	10.01
c	Physcion	Non-Competitive inhibition	94.75
d	Rhein	Mixed Type inhibition	127.3
e	Citreorosein	Mixed Type inhibition	18.56
f	Emodin-8-O-glc	Competitive inhibition	3.425
g	Polygonumnolide C2	Non-Competitive inhibition	4.291
h	Polygonumnolide C3	Non-Competitive inhibition	12.89
i	Polygonumnolide C4	Un-Competitive inhibition	77.42
j	Trans-emodin dianthrones	Competitive inhibition	1.083
k	Cis-emodin dianthrones	Competitive inhibition	0.8630

### Structure–activity relationships

The structure–activity relationships of the tested compounds were evaluated. Emodin, physcion, rhein, citreorosein, and emodin-8-O-glu have the same skeleton type with different substituents (Fig. 1). Among them, emodin exhibited moderate inhibitory activity against UGT1A1 (K_i_ = 10.01 μM). Introduction of a hydroxyl group at the 4-position resulted in slightly decreased activity (K_i_ = 18.56 μM for citreorosein), probably due to formation of an intramolecular hydrogen bond with the 5-carbonyl group, which hampers binding to UGT1A1. Besides, hydration between the additional hydroxyl groups and the water molecules present in the enzyme might also prevent their interaction with the residues in the binding pocket that would be another cause of the decreased activity, which requires further investigation.

Additionally, 8-Glycosylation of emodin markedly enhanced its inhibitory potency (K_i_ = 3.425 μM for emodin-8-O-glu), probably due to the presence of multiple hydroxyl groups on the glucose moiety, providing more sites for binding to UGT1A1. However, rhein (K_i_ = 127.3 μM) and physcion (K_i_ = 94.75 μM) showed extremely weak affinity for UGT1A1, for which two explanations are possible: First, compared with emodin, the 6-hydroxyl groups of rhein and physcion were methylated or absent, and rhein has an additional carboxyl group at C-3 that may affect its interaction with UGT1A1. Second, because the structures of rhein and physcion are similar to that of emodin, whereas their activity and mode of inhibition differ markedly, the former two compounds may bind to other sites of UGT1A1.

Compounds other than those mentioned above were emodin-type dianthrone derivatives (Fig. 1), which can be synthesized from two molecules of emodin via enzyme-catalyzed reduction and intermolecular oxidative coupling reactions^42^. Both trans- and cis-emodin dianthrones exhibited strong inhibitory effects on UGT1A1 (K_i_ = 1.083 and 0.863 μM, respectively). The two π systems and multiple hydroxyl groups of these two compounds may increase their affinity for UGT1A1 by facilitating hydrophobic interactions and hydrogen bonding.

Because of their large volume and rigidity, the spatial orientation of the tested compounds, which is dependent mainly on the configurations of C10 and C10′, influences their binding to UGT1A1. Unfortunately, the trans- and cis-emodin dianthrones tested in this study were racemic mixtures; thus, the relative contributions of the individual pure optical isomers to their inhibitory activities against UGT1A1 are unclear.

Polygonumnolides C2-C4 are C10/C10′ diastereomers of emodin dianthrone-8-O-β-d-glucopyranoside (Fig. 1). The presence of a glucose unit further increases the molecular volume, and the resulting intramolecular hydrogen bonding with the aglycone may stabilize the molecular conformation and thereby enhance their binding stereo-selectivity with UGT1A1, resulting in markedly different levels of inhibition of UGT1A1 (K_i_ = 4.219 μM for C2, 12.89 μM for C3, and 77.42 μM for C4).

### Analysis of the interactions between *P. multiflorum* compounds and UGT1A1

To investigate the mechanism underlying the UGT1A1 inhibitory activities of the *P*. *multiflorum* compounds, molecular modeling studies were performed (Supplementary Table S1).

**Figure 2:**
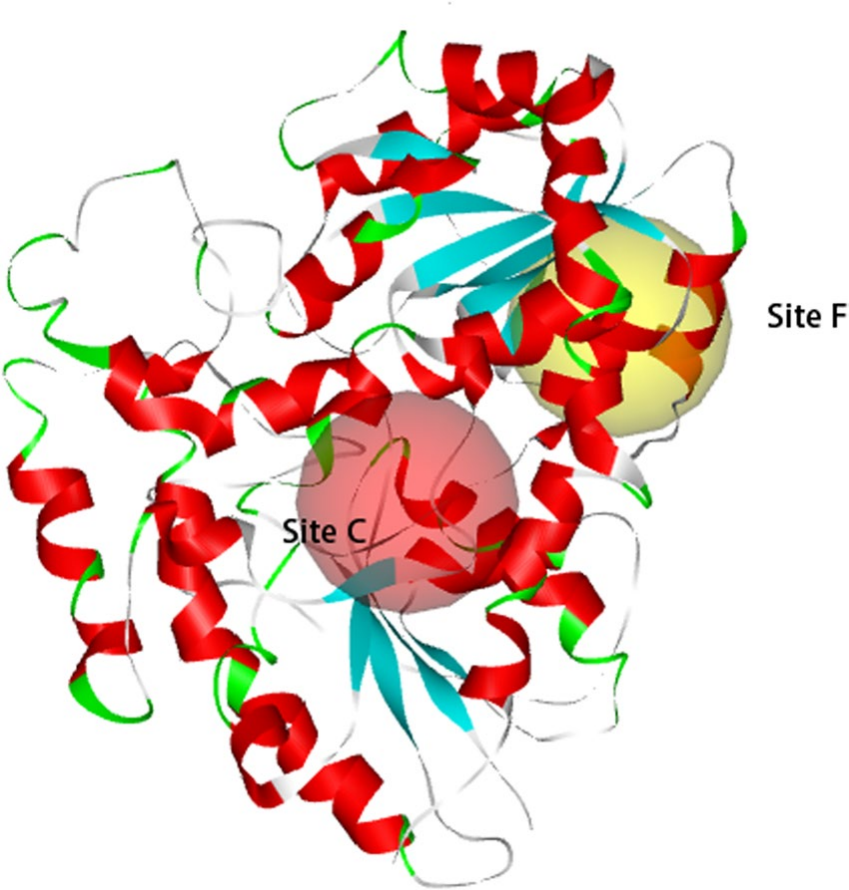
The active site C and F in UGT1A1.

Nine active sites of UGT1A1 were identified using the From Receptor Cavities module of Discovery Studio 2.5. The coordinates and radii of the active sites are shown in Supplementary Table S2. All compounds docked into active sites C and F (Fig. 2), depending on their mode of inhibition (Table 1). Emodin, cis-emodin dianthrones, trans-emodin dianthrones, and emodin-8-O-glc showed competitive inhibition, which is consistent with both the docking of the bilirubin substrate into active site F and the *in vitro* inhibition data. The other compounds exhibited non-competitive, mixed-competitive, and un-competitive inhibition and docked primarily into active site C.

**Figure 3:**
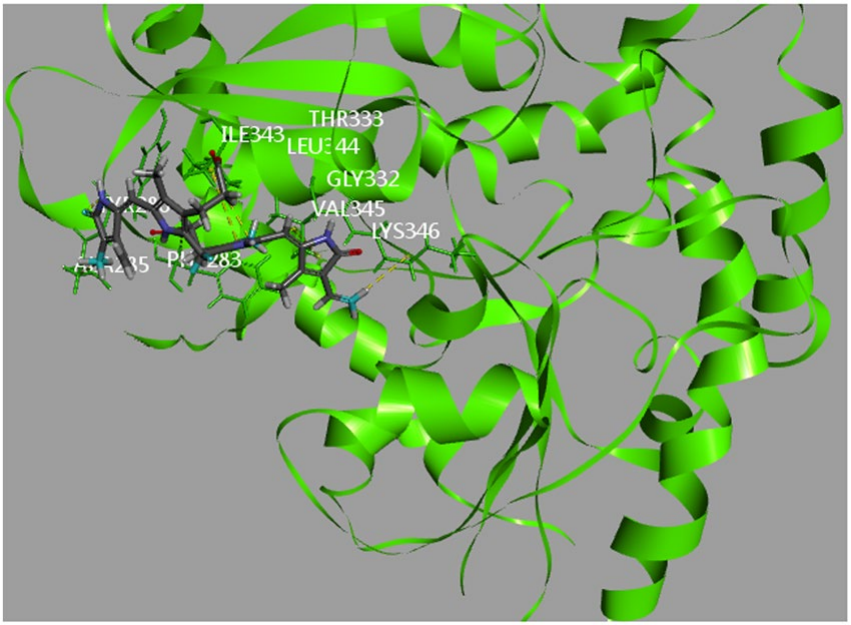
Computational docking of bilirubin into the active sites of UGT1A1.

The molecular modeling results revealed that the compounds exhibit hydrophobic interactions with active site F of UGT1A1. These interactions were similar to those between bilirubin and UGT1A1 (Fig. 3).

**Figure 4:**
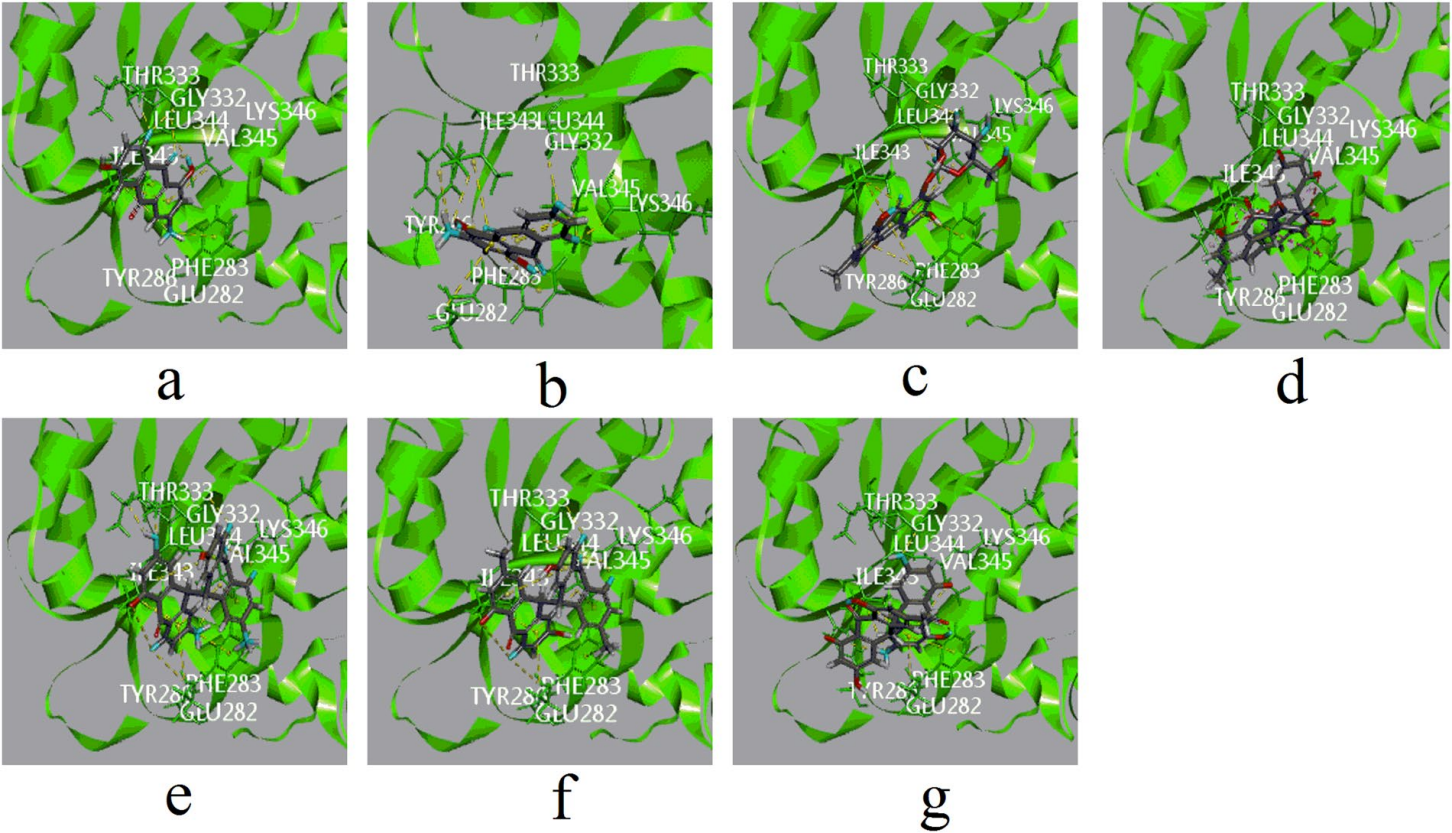
Computational docking of ligands in site F. The interaction between ligands and amino acid residues of UGT1A1 (**a**) emodin with ILE343, VAL345, LEU344, THR333 and GLY332. (**b**) Citreorosein with ILE343, VAL345, PHE283, LYS346 and GLY332. (**c**) Emodin-8-O-glc with ILE343, VAL345, PHE283, LYS346, GLY332 and LEU344. (**d**) Trans-emodin dianthrone (10αH/10βH) with VAL345, ILE343 and LY332. (**e**) Trans-emodin dianthrone (10 βH/10αH) with VAL345, ILE343, GLY332, THR333, LYS346 and VAL345 (**f**) cis-emodin dianthrone (10αH/10αH) with VAL345, ILE343, GLY332, LYS346 and VAL345 (**g**) cis-emodin dianthrone (10βH/10βH) with PHE283, VAL345, ILE343 and GLY332). All involved ligands and side chains by element had been coloured.

Among the emodin-type anthraquinones, the skeletons of cierosein and emodin-8-O-glc displayed π-alkyl hydrophobic interactions similar to those of emodin with ILE343 and VAL345. Moreover, these two compounds showed a π-π T-shaped hydrophobic interaction with PHE283, which served to stabilize the complexes (Fig. 4).

In addition to hydrophobic interactions, the hydroxyl groups at the 1- and 8-positions, as well the carbonyl group at the 9-position, of emodin formed hydrogen bonds with LEU344, THR333, and GLY332, respectively. Compared to emodin, the sugar moiety of emodin-8-O-glc formed three additional hydrogen bonds with LYS346, GLY332, and LEU344, although the hydrogen bonds between the aglycone and amino groups were weak. Cierosein displayed a similar bonding mode to emodin; however, the extra hydroxyl group at the 4-position was not involved in interactions with amino acid residues.

The CDOCKER interaction energies of emodin, cierosein, and emodin-8-O-glc were −25.99932, −29.6367, and −34.7005 kcal/mol, respectively. These values are not completely in accordance with the inhibition activity ranks: emodin-8-O-glc > emodin > cierosein. As the crystal structure of UGT1A1 has not been determined, in the homology model water molecules were removed for the position of water molecules could not been determined. It is possible that the additional hydroxyl group in citreorose results in increased hydration and a weaker interaction between citreorosein and UGT1A1.

In active site F, due to their larger conjugated system, the dianthrone derivatives of cis-emodin dianthrones and trans-emodin dianthrones showed several π-alkyl and π-π T-shaped hydrophobic interactions with PHE283, VAL345, and ILE343. Therefore, the hydrophobic interactions of these compounds were much more than those of the emodin-type anthraquinones.

As cis-emodin dianthrones and trans-emodin dianthrones are racemic mixtures, different configurations of 10 and 10′ affect their space configurations, and the increased molecular volume enhances rigidity, which alters their docking orientations. Trans-emodin dianthrone (10βH/10αH) and cis-emodin dianthrone (10αH/10αH) formed six and five hydrogen bonds with GLY332, THR333, LYS346, and VAL345, respectively, which resulted in low active site C docking energies of −42.4254 and −39.8091 kcal/mol.

In addition to hydrophobic interactions, cis-emodin dianthrone (10βH/10βH) and trans-emodin dianthrone (10αH/10βH) formed a hydrogen bond with GLY332, which may explain their weaker bonding force with active site F compared with their racemates. The binding energies of cis-emodin dianthrone (10βH/10βH) and trans-emodin dianthrone (10αH/10βH) were −28.2228 and −25.4912 (kcal/mol), respectively. However, these results were obtained using racemic mixtures of the test compounds.

**Figure 5:**
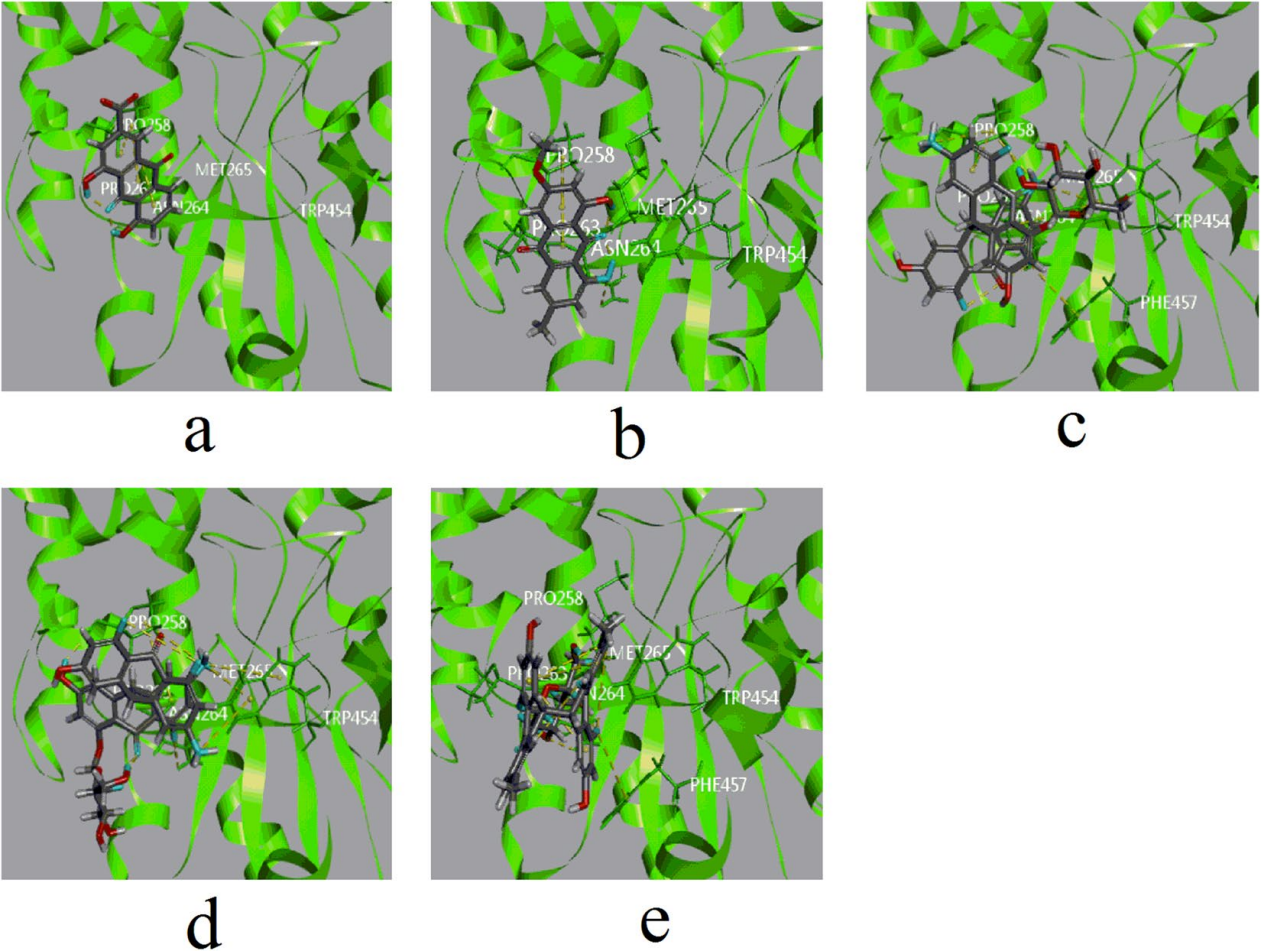
Computational docking of ligands in site C. The interaction between ligands and amino acid residues of UGT1A1 (**a**), rhein with PRO258 ASN264 (**b**), physcion with PRO258 (**c**) polygonumnolide C2 withTRP454, PRO258 and ASN264 (**d**) polygonumnolide C3 with TRP454, PRO258 and ASN264 (**e**) polygonumnolide C4 with ASN264, MET265 and PRO263). All involved ligands and side chains by element had been coloured.

In active site C (Fig. 5), rhein and physcion showed docking energies of −20.6433 and −19.7442 kcal/mol, respectively, and showed π-alkyl hydrophobic interactions with PRO258. Rhein showed no other interactions with surrounding amino acid residues, which may explain its weaker bonding with UGT1A1. The carboxyl group of rhein may induce hydration, especially in the absence of counterpart basic residues in the binding pocket, which could also be responsible for its relatively weak inhibition of UGT1A1 *in vitro*.

The molecular diameters of polygonumnolides C2–4 are 7.5, 8.2, and 8 Å, respectively, which are similar to the size of active site C (7.9 Å). A larger molecular volume leads to enhanced rigidity, which may affect the docking orientation.

No hydrophobic interactions, but six hydrogen bonds with ASN264, MET265, and PRO263, as well as seven intramolecular hydrogen bonds, between polygonumnolide C4 and amino acid residues, were detected. The binding energy of polygonumnolide C4 (−31.1608 kcal/mol) suggested that its relative lack of hydrophobic interactions might led to low affinity for UGT1A1.

The binding modes of polygonumnolides C2 and C3 (π-alkyl hydrophobic interactions with TRP454 and PRO258) were different from those of polygonumnolide C4. Furthermore, polygonumnolide C2 formed an additional tight π-π stacked hydrophobic interaction with TRP454. Moreover, polygonumnolides C2 and C3 were stabilized by hydrogen bonding with ASN264 and PRO258. The CDOCKER interaction energies of polygonumnolide C2 and C3 (−36.9558 and −37.2953 kcal/mol, respectively) suggested that these compounds have high affinity for UGT1A1.

Computational docking studies were performed to evaluate the interactions of the tested compounds with UGT1A1. The results suggested that tight hydrophobic interactions are crucial for the affinity between ligands and UGT1A1. The formation of hydrogen bonds via hydroxyl groups enhanced the UGT1A1-binding affinity of the tested compounds.

## Discussion

Quinones are widely distributed in plant species and have multiple pharmacological activities^43^. However, some quinines inhibit UGTs, leading to adverse reactions. For example^44^, OTS167, an antineoplastic agent, is metabolized mainly by UGT1A1 and UGT1A3, and is inhibited by emodin (an inhibitor of UGT1A8 and UGT1A10). At the concentrations required to exert an antitumor effect, celastrol interacts with herbs and drugs^45^. Therefore, determination of the UGT inhibitory effects of quinone compounds is required to enable prediction of drug–drug and herb–drug interactions.

In this study, we evaluated the inhibition of UGT1A1 by a *P*. *multiflorum* extract and 10 compounds with diverse structures. The *P*. *multiflorum* extract and several of its anthraquinone and anthrone components inhibited UGT1A1 activity. The *P*. *multiflorum* extract exhibited a greater inhibitory effect than any of the individual components. The postulated biogenetic relationship among emodin, emodin glucopyranoside, emodin dianthrones (trans-emodin dianthrones and cis-emodin dianthrones), and polygonumnolides C2-C4 may underlie the UGT1A1 inhibitory activity of the *P*. *multiflorum* extract^42^. As the *P*. *multiflorum* extract comprises a large number of compounds, the identity and character of the active components should be investigated.

We conducted a mechanistic analysis by the molecular docking method. As the structure of the N-terminal domain of UGT1A1 has not been determined, the crystal structures of four proteins were used as templates. The results were generally consistent with the *in vitro* inhibition data and are in agreement with previous reports that UGT1A1 has at least two binding sites for xenobiotics and endobiotics^46,47^.

The compounds evaluated in this study had an anthraquinone nucleus structure. The molecular modeling indicated that the presence of large conjugated systems (*e*.*g*., the dianthronoid derivatives) enhanced hydrophobic interactions with amino acid residues of UGT1A1. However, the large molecular structure of dianthrone and dianthrone glycosides resulted in increased rigidity. The spatial configuration, particularly the 10 or 10′ configuration, of a molecule with a diameter equal to or greater than that of the active pocket affects its hydrophobic interactions with amino acid residues.

In conclusion, to our knowledge, this study is the first to evaluate the effects of representative quinone constituents of *P*. *multiflorum* on UGT1A1 activity *in vitro* and to examine their structure–activity relationships. Notably, cis-emodin dianthrones, trans-emodin dianthrones, and emodin-8-O-glc showed strong inhibition of UGT1A1 and thus warrant particular attention. Moreover, the findings enhance our understanding of the mechanisms underlying *P*. *multiflorum* toxicity and will facilitate further studies of UGT1A1-mediated drug–drug interactions in patients taking *P*. *multiflorum*.

## Materials and Methods

### Reagents and materials

Bilirubin (99.3%), Trizma base, and alamethicin were obtained from J&K Scientific (Beijing, China). Uridine diphosphoglucuronic acid (UDPGA), d-saccharic acid 1,4-lactone, and MgCl_2_ were purchased from Sigma-Aldrich (Beijing, China). Pooled male Sprague–Dawley RLM (452501) were obtained from BD Gentest (Shanghai, China). Emodin (99.3%), physcion (99.2%), and rhein (99.8%) were purchased from the National Institute for Food and Drug Control (Beijing, China). Emodin-8-O-glc (98.0%), citreorosein (98.0%), trans-emodin dianthrones (98.0%), and cis-emodin dianthrones (98.0%) were purchased from Shanghai Yuanye Bio-Technology Co. (Shanghai, China). Polygonumnolides C2, C3, and C4 and the *P*. *multiflorum* extract were gifts from Dr. Yang (Institute of Materia Medica, Chinese Academy of Medical Sciences & Peking Union Medical College). Polygonumnolides C2, C3, and C4 were isolated and characterized by Dr. Yang and colleagues^20^. All other chemicals used in the glucuronidation incubations and the ultra-performance liquid chromatography (UPLC) solvents were of high-performance LC grade and were obtained from Sigma-Aldrich (Beijing, China).

### Preparation of the *P. multiflorum* extract

Dried roots of *P*. *multiflorum* (28.0 kg) were extracted three times with 70% ethanol. The extracts were concentrated under reduced pressure at <50 °C, and the resulting concentrated extract (4.0 kg) was used in subsequent experiments.

### Ultra-performance liquid chromatography

We previously established a complete ultra-performance liquid chromatography (UPLC) method to detect bilirubin glucuronides for the purpose of analyzing the kinetic parameters of UGT1A1^41^. Chromatographic analyses were carried out using an Acquity UPLC system (Waters, Milford, MA, USA) equipped with a binary pump, automatic sampler, photo-diode array detector, system controller, and temperature-controlled oven. Bilirubin and its glucuronides were separated on an Acquity UPLC HSS C18 column (2.1 × 100 mm; 1.8 μm) with a guard column (Acquity UPLC HSS C18 VanGuard pre-column, 2.1 × 5 mm, 1.8 μm). The mobile phase consisted of 0.1% formic acid in water (A) and 100% acetonitrile (B) and was delivered at a flow rate of 0.4 mL/min. The linear gradient elution program was as follows: 0–2.1 min, 40–75% B; 2.1–4.2 min, 75–95% B; 4.2–8.0 min, 95% B; 8.0–8.5 min, 95–40% B. The column temperature was 35 °C. The detection wavelength was 450 nm, and the sample injection volume was 10 μL.

### Assay of UGT1A1 activity

UGT1A1 activity assays were conducted at 37 °C in a shaking water bath^41^. Incubation mixtures (final volume, 0.2 mL) contained 0.5 mg/mL RLM, 0.1 M Tris-HCl (pH 7.4), 5 mM MgCl_2_, 5 mM d-saccharic acid 1,4-lactone, 3.5 mM UDPGA, and 50 mg/g protein alamethicin. Bilirubin (as a UGT1A1 substrate) and the test samples were dissolved in 100% dimethylsulfoxide (DMSO) immediately before being added to the incubation mixtures. The final DMSO concentration was ≤1%, because preliminary experiments indicated that higher concentrations affect the metabolic activity of liver microsomes. The reaction was initiated by adding UDPGA after a 5-min pre-incubation at 37 °C; it was terminated after 10 min by adding 0.6 mL of ice-cold methanol: acetonitrile (1:2) containing 200 mM ascorbic acid. Proteins were precipitated by centrifugation at 13,000 × *g* for 30 min at 4 °C prior, and the supernatants were analyzed by UPLC.The assays were done in triplicates respectively.

### Determination of enzyme inhibition kinetics

Bilirubin glucuronidation was evaluated in the presence of various concentrations of bilirubin and potential inhibitors, as described above. Samples were analyzed in triplicate to determine their inhibition constants (K_i_). For assays with inhibitors, the bilirubin concentration was 0.12 to 1.37 μM. The concentration ranges of the potential inhibitors were as follows: emodin, 0.36–11.6 μM; physcion, 0.15–9.3 μM; rhein, 0.35–11 μM; emodin-8-O-glc, 0.02–3.4 μM; citreorosein, 1.45–11.6 μM; trans-emodindianthrones, 0.018–0.29 μM; cis-emodindianthrones, 0.015–0.25 μM; polygonumnolide C2, 0.08–1.3 μM; polygonumnolide C3, 0.15–2.5 μM; polygonumnolide C4, 0.016–0.26 μM; and *P*. *multiflorum* extract, 1.5–25 μM (because the extract was a mixture, this concentration was based on the highest concentration of emodin).

The inhibition kinetic type and parameters were determined by fitting the reaction velocities of different concentrations of test components (inhibitors) and bilirubin (substrate). Lineweaver–Burk and Dixon fitting equations were employed to determine the inhibition type, and the second plot (drawn using the slope of lines in the Lineweaver–Burk plot versus inhibitor concentrations) was used to calculate the K_i_ values. The results were analyzed using GraphPad Prism 5 software (GraphPad Inc., San Diego, CA, USA)^48^. The mean kinetic constants (K_m_, V_max_, and K_i_) ± standard errors are reported. The goodness-of-fit of the inhibition models was estimated using the F statistic, R^2^ value, parameter standard error estimates, and 95% confidence intervals^41^. The assays were done in triplicates respectively (Supplementary Fig. S1).

### Computational modeling

Because the structure of the N-terminal domain of UGT1A1 has not been determined, the properties of the active site of UGT1A1 are unclear. In this study, the BLAST search module of Discovery Studio 2.5 was used to compare amino acid sequence identities and similarities. The following four proteins were selected as templates for homology modeling by the BLAST module: UDP-glucuronosyltransferase 2B7 (PDB: 2O6L), UDP-glucuronosyl/UDP-glucosyltransferase (PDB: 2PQ6), UDP-glucose flavonoid 3-O glycosyltransferse (PDB: 2C1X), and oleandomycin glycosyltransferase (PDB: 2IYA)^49,50^. The similarities were 72%, 43%, 46%, and 54%, respectively, and the identities were 56%, 28%, 27%, and 34%, respectively. The homology models were built using Modeller 9.13. Five candidate models for further optimization were generated by the multi-template modeling method. In this study, all heavy atoms were constrained, the hydrogen atoms were optimized, and the side chains and the loop region were refined based on the energy minimization method, which used the steepest descent method and the conjugate gradient method^51^. Finally, the homology models were evaluated by generating a Ramachandran plot and Profile-3D analysis^52^. The optimum amount of amino acid residues was 96.37%, and the verify score was 156.27. The verify score suggested that the model structure had good three-dimensional chemical parameters and spatial structure. The CDOCKER interaction energies were used to evaluate the molecular protein–ligand interactions^53^_._

## Electronic supplementary material

supporting information
