# Supplementary material for: Identification and characterization of the structure–activity relationships involved in UGT1A1 inhibition by anthraquinone and dianthrone constituents of Polygonum multiflorum
Source: Sci Rep. 2017 Dec 20;7:17952. doi: 10.1038/s41598-017-18231-y (PMC5738440; doi:10.1038/s41598-017-18231-y)
Supplement: Supplementary file 1 — supporting information [file 41598_2017_18231_MOESM1_ESM.doc]

**Identification and characterization of the structure–activity relationships involved inUGT1A1 inhibition by anthraquinone and dianthrone constituents of *Polygonum multiflorum***

Qi Wang^1, 2^, YadanWang^2^, Yong Li^1^, Binyu Wen^3^, Zhong Dai^2^, ShuangchengMa^1, 2^*, YujieZhang^1^*

*^1^Beijing University of Chinese Medicine*

*^2^National Institutes for Food and Drug Control*

*^3^Dongfang Hospital, Beijing University of Chinese Medicine*

Figure S1


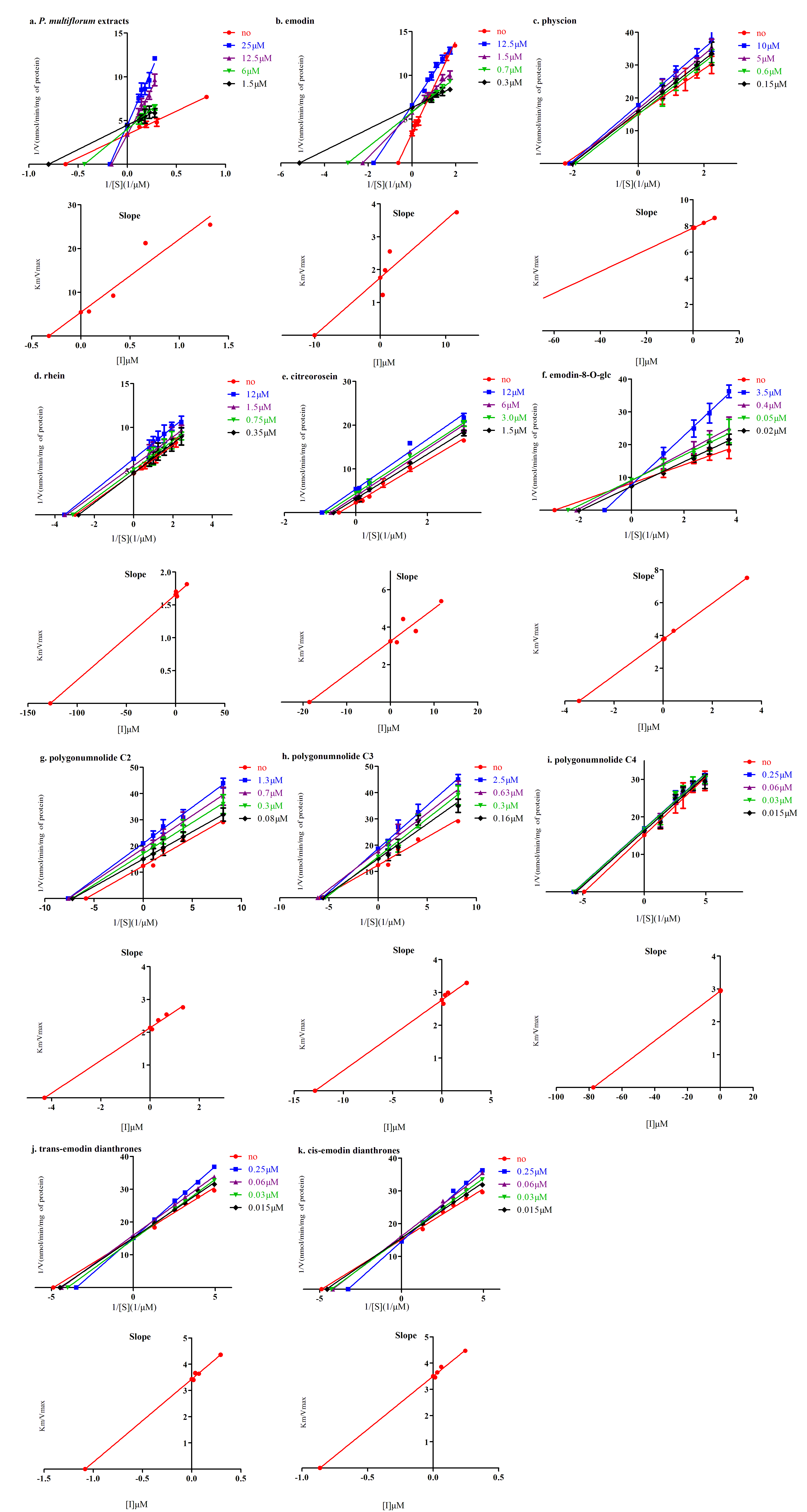


Figure S1. Inhibition kinetics of *P. multiflorum* extracts and its individual components towards UGT1A1-catalysed glucuronidation of bilirubin. Lineweaver–Burk and Dixon fitting equations were employed to determine the inhibition type, and the second plot (drawing using the slope of lines from Lineweaver–Burk plot versus the concentrations of inhibitors) was used to calculate the inhibition kinetic parameters (*K_i_*). Data represent the mean ± standard deviation of triplicate samples.

**Table S**1. Interaction studies of ligands and UGT1A1.

| **Compound A. Trans-emodin dianthrone (10αH/10βH)**  **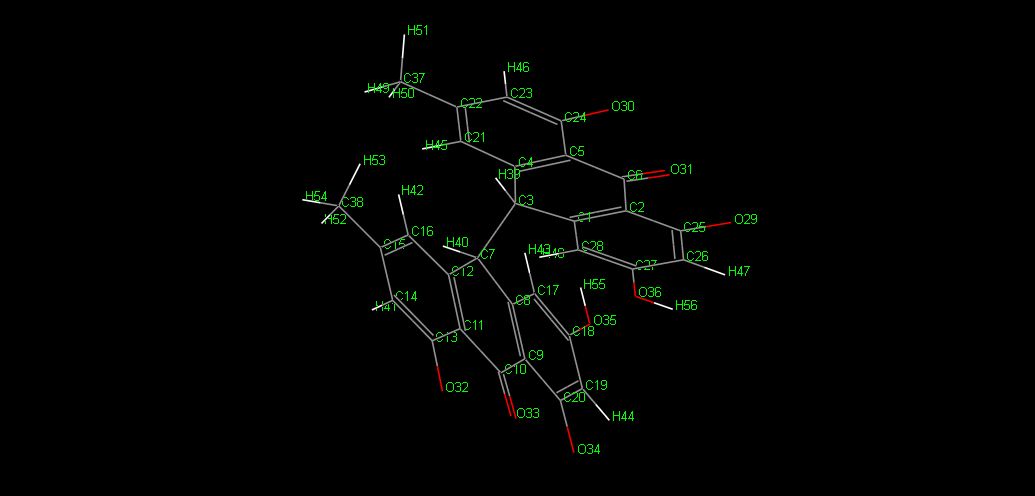** | | | | | | | |
| --- | --- | --- | --- | --- | --- | --- | --- |
| Name | Distance | CatCory | Types | From | From-Chemistry | to | To-Chemistry |
| :GLY332:HA1 - A:O35 | 2.321 | Hydrogen Bond | Carbon Hydrogen Bond | :GLY332:HA1 | H-Donor | A:O35 | H-Acceptor |
| A:O32 - :PHE283 | 3.11106 | Electrostatic | Pi-Anion | A:O32 | Negative | :PHE283 | Pi-Orbitals |
| :PHE283 - A | 5.11864 | Hydrophobic | Pi-Pi T-shaped | :PHE283 | Pi-Orbitals | A | Pi-Orbitals |
| A:C38 - :ILE343 | 4.14373 | Hydrophobic | Alkyl | A:C38 | Alkyl | :ILE343 | Alkyl |
| :TYR286 - A:C38 | 4.31105 | Hydrophobic | Pi-Alkyl | :TYR286 | Pi-Orbitals | A:C38 | Alkyl |
| A - :ILE343 | 4.31998 | Hydrophobic | Pi-Alkyl | A | Pi-Orbitals | :ILE343 | Alkyl |
| A - :VAL345 | 4.98844 | Hydrophobic | Pi-Alkyl | A | Pi-Orbitals | :VAL345 | Alkyl |
| **Compound B. Trans-emodin dianthrone (10βH/10α H)**  **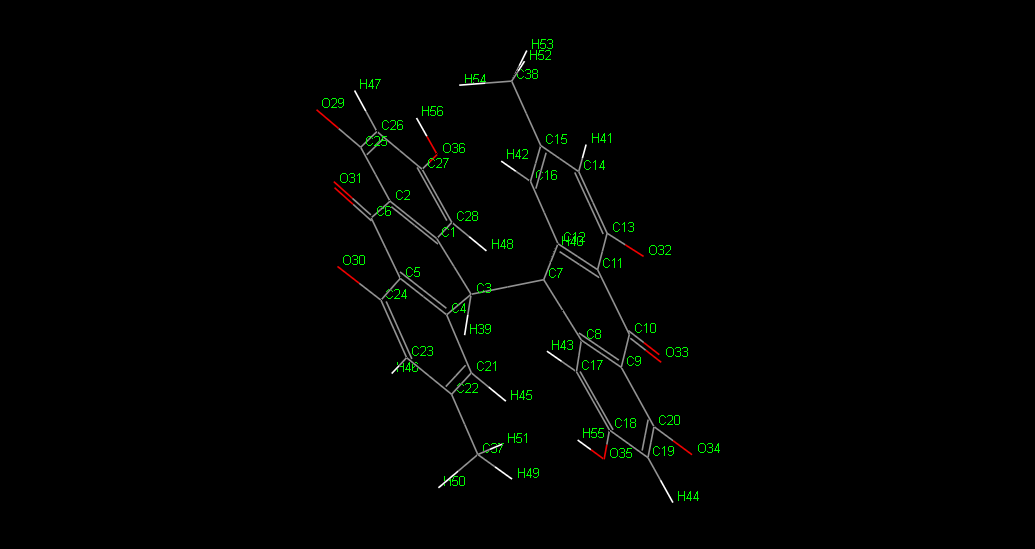** | | | | | | | |
| :GLY332:HN - B:O29 | 2.71703 | Hydrogen Bond | Conventional Hydrogen Bond | :GLY332:HN | H-Donor | B:O29 | H-Acceptor |
| :THR333:HN - B:O35 | 1.91464 | Hydrogen Bond | Conventional Hydrogen Bond | :THR333:HN | H-Donor | B:O35 | H-Acceptor |
| :LYS346:HN - B:O30 | 1.90918 | Hydrogen Bond | Conventional Hydrogen Bond | :LYS346:HN | H-Donor | B:O30 | H-Acceptor |
| :LYS346:HN - B:O31 | 2.73791 | Hydrogen Bond | Conventional Hydrogen Bond | :LYS346:HN | H-Donor | B:O31 | H-Acceptor |
| B:H55 - :THR333:OG1 | 2.17854 | Hydrogen Bond | Conventional Hydrogen Bond | B:H55 | H-Donor | :THR333:OG1 | H-Acceptor |
| :VAL345:HA - B:O31 | 2.62567 | Hydrogen Bond | Carbon Hydrogen Bond | :VAL345:HA | H-Donor | B:O31 | H-Acceptor |
| :GLU282:OE2 - B | 4.71386 | Electrostatic | Pi-Anion | :GLU282:OE2 | Negative | B | Pi-Orbitals |
| B - B | 4.39181 | Hydrophobic | Pi-Pi Stacked | B | Pi-Orbitals | B | Pi-Orbitals |
| B:C38 - :ILE343 | 5.08053 | Hydrophobic | Alkyl | B:C38 | Alkyl | :ILE343 | Alkyl |
| B:C38 - :VAL345 | 3.87102 | Hydrophobic | Alkyl | B:C38 | Alkyl | :VAL345 | Alkyl |
| :PHE283 - B:C37 | 4.72697 | Hydrophobic | Pi-Alkyl | :PHE283 | Pi-Orbitals | B:C37 | Alkyl |
| :PHE283 - B:C38 | 3.45878 | Hydrophobic | Pi-Alkyl | :PHE283 | Pi-Orbitals | B:C38 | Alkyl |
| B - :VAL345 | 4.58857 | Hydrophobic | Pi-Alkyl | B | Pi-Orbitals | :VAL345 | Alkyl |
| B - :ILE343 | 4.13926 | Hydrophobic | Pi-Alkyl | B | Pi-Orbitals | :ILE343 | Alkyl |
| B - :VAL345 | 5.49783 | Hydrophobic | Pi-Alkyl | B | Pi-Orbitals | :VAL345 | Alkyl |
| **Compound C. Cis-emodin dianthrone (10αH/10αH)**  **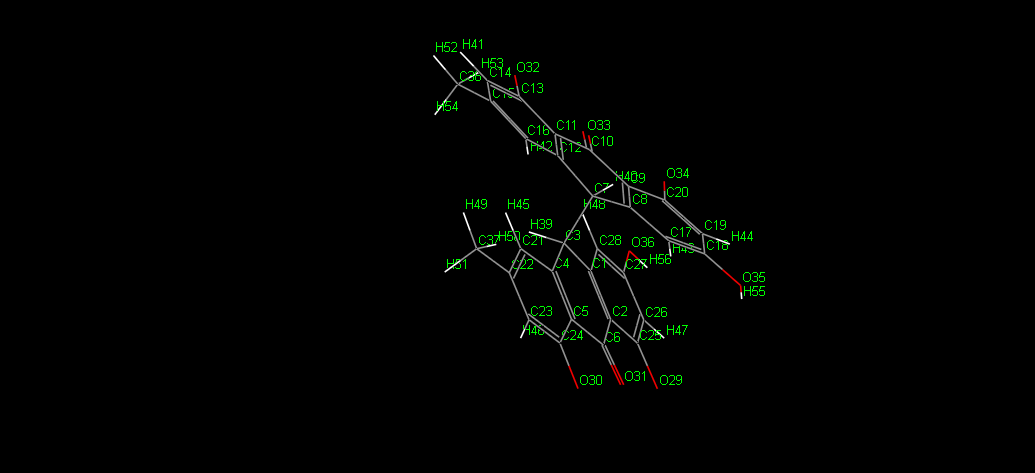** | | | | | | | |
| :GLY332:HN - C:O34 | 2.8748 | Hydrogen Bond | Conventional Hydrogen Bond | :GLY332:HN | H-Donor | C:O34 | H-Acceptor |
| :LYS346:HN - C:O32 | 1.92242 | Hydrogen Bond | Conventional Hydrogen Bond | :LYS346:HN | H-Donor | C:O32 | H-Acceptor |
| :LYS346:HN - C:O33 | 2.91019 | Hydrogen Bond | Conventional Hydrogen Bond | :LYS346:HN | H-Donor | C:O33 | H-Acceptor |
| :GLY332:HA2 - C:O33 | 3.08562 | Hydrogen Bond | Carbon Hydrogen Bond | :GLY332:HA2 | H-Donor | C:O33 | H-Acceptor |
| :VAL345:HA - C:O33 | 2.65685 | Hydrogen Bond | Carbon Hydrogen Bond | :VAL345:HA | H-Donor | C:O33 | H-Acceptor |
| :GLU282:OE2 - C | 4.89779 | Electrostatic | Pi-Anion | :GLU282:OE2 | Negative | C | Pi-Orbitals |
| C - C | 4.47651 | Hydrophobic | Pi-Pi Stacked | C | Pi-Orbitals | C | Pi-Orbitals |
| :PHE283 - C:C38 | 4.78266 | Hydrophobic | Pi-Alkyl | :PHE283 | Pi-Orbitals | C:C38 | Alkyl |
| C - :ILE343 | 4.19694 | Hydrophobic | Pi-Alkyl | C | Pi-Orbitals | :ILE343 | Alkyl |
| C - :VAL345 | 5.15441 | Hydrophobic | Pi-Alkyl | C | Pi-Orbitals | :VAL345 | Alkyl |
| C - :ILE343 | 5.48434 | Hydrophobic | Pi-Alkyl | C | Pi-Orbitals | :ILE343 | Alkyl |
| C - :VAL345 | 4.5997 | Hydrophobic | Pi-Alkyl | C | Pi-Orbitals | :VAL345 | Alkyl |
| **Compound D. Cis-emodin dianthrone (10βH/10βH)**  **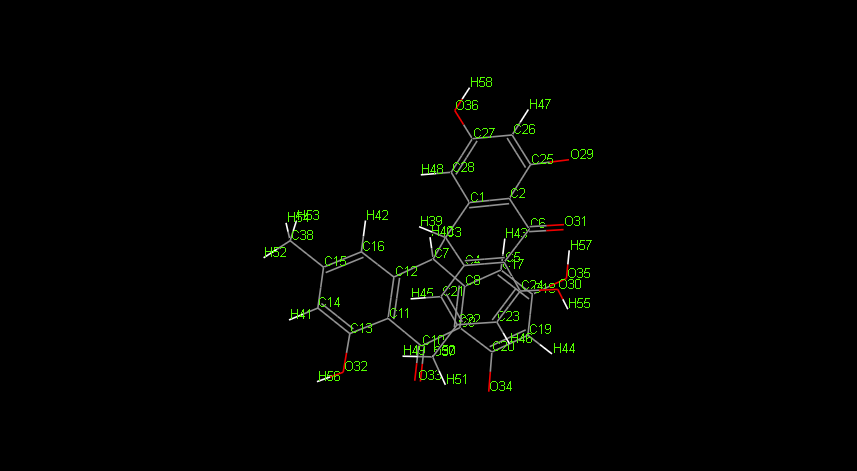** | | | | | | | |
| D.cdx:H55 - D.cdx:O31 | 2.01216 | Hydrogen Bond | Conventional Hydrogen Bond | D.cdx:H55 | H-Donor | D.cdx:O31 | H-Acceptor |
| D.cdx:H56 - D.cdx:O33 | 1.91288 | Hydrogen Bond | Conventional Hydrogen Bond | D.cdx:H56 | H-Donor | D.cdx:O33 | H-Acceptor |
| :GLY332:HA1 - D.cdx:O35 | 2.34784 | Hydrogen Bond | Carbon Hydrogen Bond | :GLY332:HA1 | H-Donor | D.cdx:O35 | H-Acceptor |
| :GLU282:OE2 - D.cdx | 4.64904 | Electrostatic | Pi-Anion | :GLU282:OE2 | Negative | D.cdx | Pi-Orbitals |
| :PHE283 - D.cdx | 5.25679 | Hydrophobic | Pi-Pi T-shaped | :PHE283 | Pi-Orbitals | D.cdx | Pi-Orbitals |
| D.cdx:C38 - :ILE343 | 4.12502 | Hydrophobic | Alkyl | D.cdx:C38 | Alkyl | :ILE343 | Alkyl |
| :TYR286 - D.cdx:C38 | 4.27095 | Hydrophobic | Pi-Alkyl | :TYR286 | Pi-Orbitals | D.cdx:C38 | Alkyl |
| D.cdx - :ILE343 | 4.53972 | Hydrophobic | Pi-Alkyl | D.cdx | Pi-Orbitals | :ILE343 | Alkyl |
| D.cdx - :VAL345 | 5.31538 | Hydrophobic | Pi-Alkyl | D.cdx | Pi-Orbitals | :VAL345 | Alkyl |
| **Compound E. Emodin-8-O-glu**  **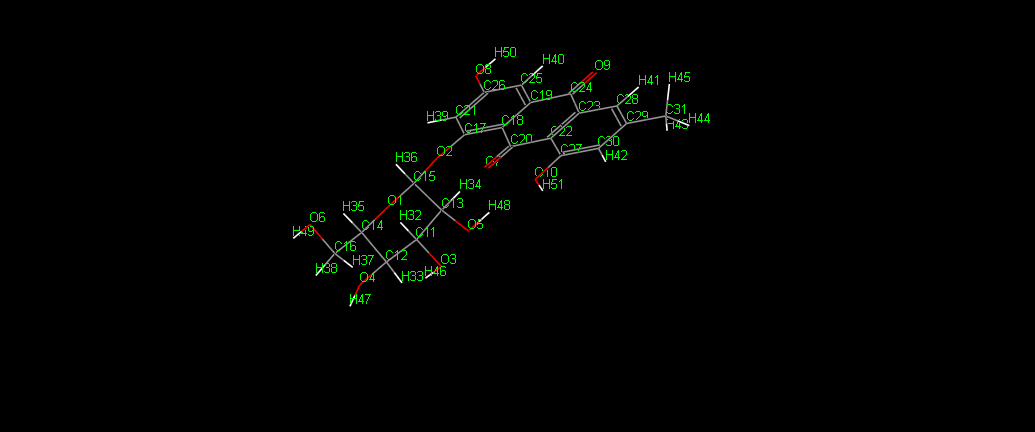** | | | | | | | |
| :LYS346:HN - E:O4 | 2.80081 | Hydrogen Bond | Conventional Hydrogen Bond | :LYS346:HN | H-Donor | E:O4 | H-Acceptor |
| E:H49 - EG:O4 | 2.08679 | Hydrogen Bond | Conventional Hydrogen Bond | E:H49 | H-Donor | E:O4 | H-Acceptor |
| E:H51 - EG:O7 | 1.88508 | Hydrogen Bond | Conventional Hydrogen Bond | E:H51 | H-Donor | E:O7 | H-Acceptor |
| :GLY332:HA1 - E:O3 | 2.56744 | Hydrogen Bond | Carbon Hydrogen Bond | :GLY332:HA1 | H-Donor | E:O3 | H-Acceptor |
| E:H36 - :LEU344:O | 2.46903 | Hydrogen Bond | Carbon Hydrogen Bond | E:H36 | H-Donor | :LEU344:O | H-Acceptor |
| :GLU282:OE2 - E | 3.52639 | Electrostatic | Pi-Anion | :GLU282:OE2 | Negative | E | Pi-Orbitals |
| :GLU282:OE2 - E | 3.56598 | Electrostatic | Pi-Anion | :GLU282:OE2 | Negative | E | Pi-Orbitals |
| :PHE283 - E | 5.56823 | Hydrophobic | Pi-Pi T-shaped | :PHE283 | Pi-Orbitals | E | Pi-Orbitals |
| E - :ILE343 | 4.43602 | Hydrophobic | Pi-Alkyl | E | Pi-Orbitals | :ILE343 | Alkyl |
| E - :VAL345 | 5.18972 | Hydrophobic | Pi-Alkyl | E | Pi-Orbitals | :VAL345 | Alkyl |
| E - :ILE343 | 4.76583 | Hydrophobic | Pi-Alkyl | E | Pi-Orbitals | :ILE343 | Alkyl |
| **Compound F. Citreorosein**  **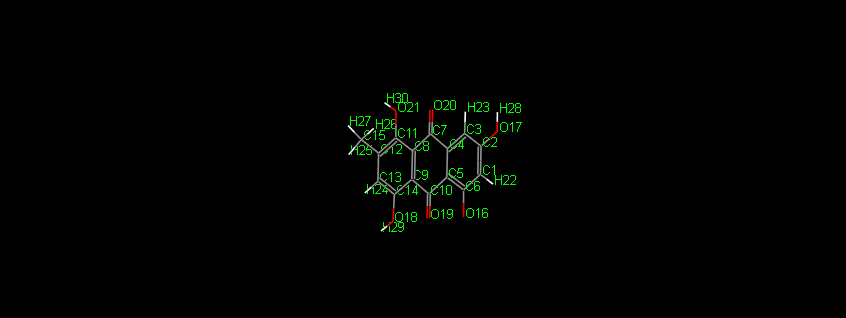** | | | | | | | |
| :LYS346:HN - F:O16 | 2.08006 | Hydrogen Bond | Conventional Hydrogen Bond | :LYS346:HN | H-Donor | F:O16 | H-Acceptor |
| F:H29 - F:O19 | 1.94479 | Hydrogen Bond | Conventional Hydrogen Bond | F:H29 | H-Donor | F:O19 | H-Acceptor |
| F:H30 - F:O20 | 1.91265 | Hydrogen Bond | Conventional Hydrogen Bond | F:H30 | H-Donor | F:O20 | H-Acceptor |
| :GLY332:HA1 - F:O17 | 2.43615 | Hydrogen Bond | Carbon Hydrogen Bond | :GLY332:HA1 | H-Donor | F:O17 | H-Acceptor |
| :VAL345:HA - F:O16 | 2.57748 | Hydrogen Bond | Carbon Hydrogen Bond | :VAL345:HA | H-Donor | F:O16 | H-Acceptor |
| :GLU282:OE2 - F | 4.57694 | Electrostatic | Pi-Anion | :GLU282:OE2 | Negative | F | Pi-Orbitals |
| :PHE283 - F | 5.7517 | Hydrophobic | Pi-Pi T-shaped | :PHE283 | Pi-Orbitals | F | Pi-Orbitals |
| F:C15 - :ILE343 | 3.92472 | Hydrophobic | Alkyl | F:C15 | Alkyl | :ILE343 | Alkyl |
| :TYR286 - F:C15 | 5.22174 | Hydrophobic | Pi-Alkyl | :TYR286 | Pi-Orbitals | F:C15 | Alkyl |
| F - :VAL345 | 4.99765 | Hydrophobic | Pi-Alkyl | F | Pi-Orbitals | :VAL345 | Alkyl |
| F - :ILE343 | 4.56596 | Hydrophobic | Pi-Alkyl | F | Pi-Orbitals | :ILE343 | Alkyl |
| F - :VAL345 | 5.30223 | Hydrophobic | Pi-Alkyl | F | Pi-Orbitals | :VAL345 | Alkyl |
| **Compound G. Emodin**  **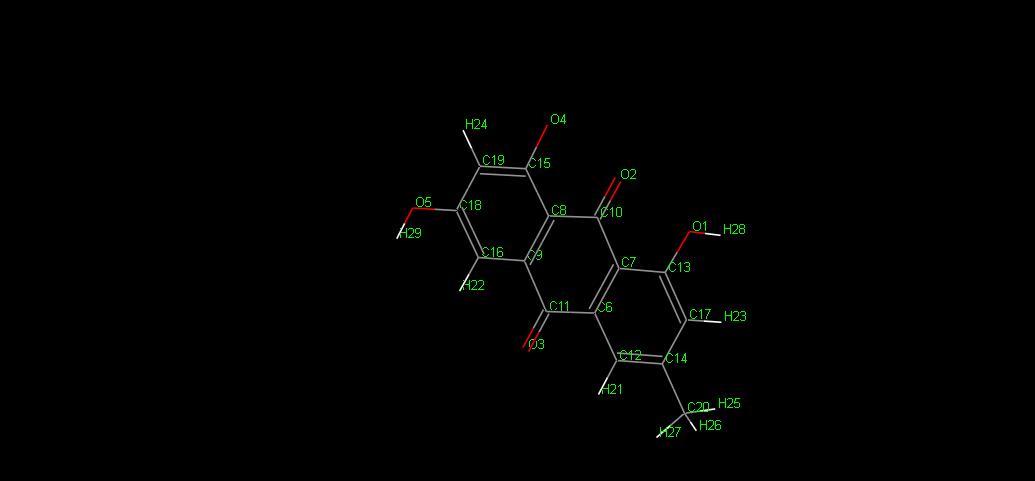** | | | | | | | |
| :THR333:HN - G:O4 | 2.0768 | Hydrogen Bond | Conventional Hydrogen Bond | :THR333:HN | H-Donor | G:O4 | H-Acceptor |
| G:H28 - :LEU344:O | 2.25358 | Hydrogen Bond | Conventional Hydrogen Bond | G:H28 | H-Donor | :LEU344:O | H-Acceptor |
| G:H28 - G:O2 | 1.87849 | Hydrogen Bond | Conventional Hydrogen Bond | G:H28 | H-Donor | G:O2 | H-Acceptor |
| :GLY332:HA1 - G:O4 | 2.4987 | Hydrogen Bond | Carbon Hydrogen Bond | :GLY332:HA1 | H-Donor | G:O4 | H-Acceptor |
| :GLY332:HA2 - G:O2 | 2.69138 | Hydrogen Bond | Carbon Hydrogen Bond | :GLY332:HA2 | H-Donor | G:O2 | H-Acceptor |
| G:C20 - :ILE343 | 4.91927 | Hydrophobic | Alkyl | G:C20 | Alkyl | :ILE343 | Alkyl |
| :PHE283 - G:C20 | 4.24455 | Hydrophobic | Pi-Alkyl | :PHE283 | Pi-Orbitals | G:C20 | Alkyl |
| G - :ILE343 | 4.28293 | Hydrophobic | Pi-Alkyl | G | Pi-Orbitals | :ILE343 | Alkyl |
| G - :ILE343 | 4.68009 | Hydrophobic | Pi-Alkyl | G | Pi-Orbitals | :ILE343 | Alkyl |
| G - :VAL345 | 5.0604 | Hydrophobic | Pi-Alkyl | G | Pi-Orbitals | :VAL345 | Alkyl |
| **Compound H. Rhein**  **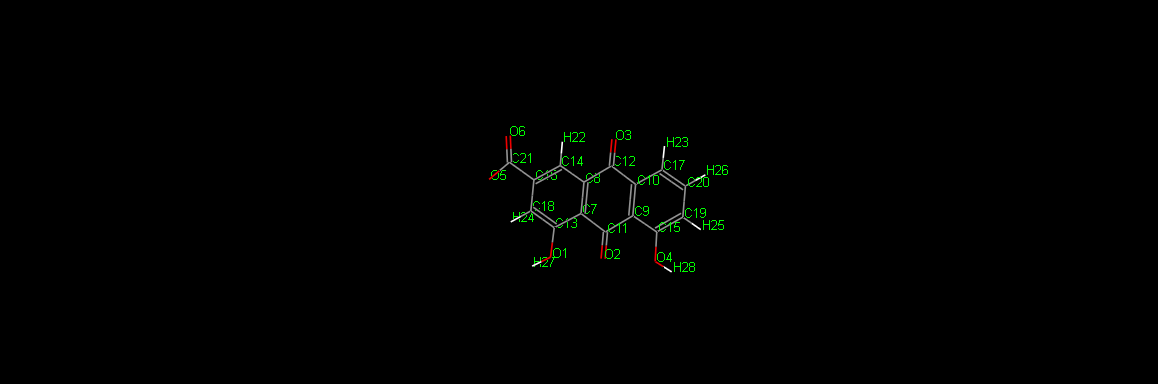** | | | | | | | |
| H:H27 - H:O2 | 1.85106 | Hydrogen Bond | Conventional Hydrogen Bond | H:H27 | H-Donor | H:O2 | H-Acceptor |
| H:H28 - H:O2 | 1.85261 | Hydrogen Bond | Conventional Hydrogen Bond | H:H28 | H-Donor | H:O2 | H-Acceptor |
| H - :PRO258 | 4.39259 | Hydrophobic | Pi-Alkyl | H | Pi-Orbitals | :PRO258 | Alkyl |
| H - :PRO258 | 4.15709 | Hydrophobic | Pi-Alkyl | H | Pi-Orbitals | :PRO258 | Alkyl |
| H - :PRO258 | 5.06508 | Hydrophobic | Pi-Alkyl | H | Pi-Orbitals | :PRO258 | Alkyl |
| **Compound I. Physcion**  **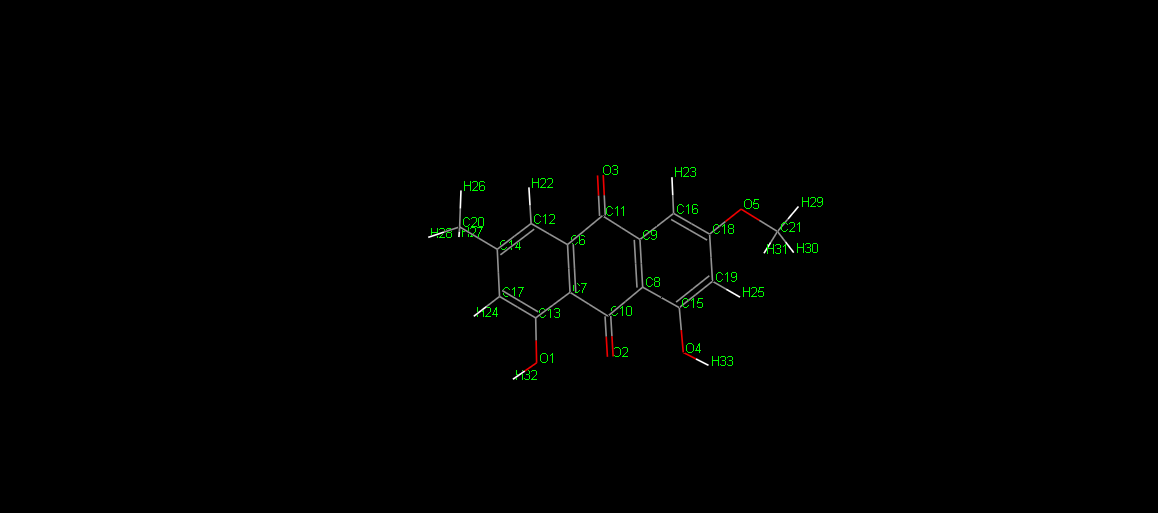** | | | | | | | |
| :ASN264:HD21 - I:O1 | 2.76591 | Hydrogen Bond | Conventional Hydrogen Bond | :ASN264:HD21 | H-Donor | I:O1 | H-Acceptor |
| I:H32 - I:O2 | 1.86423 | Hydrogen Bond | Conventional Hydrogen Bond | I:H32 | H-Donor | I:O2 | H-Acceptor |
| I:H33 - I:O2 | 1.88922 | Hydrogen Bond | Conventional Hydrogen Bond | I:H33 | H-Donor | I:O2 | H-Acceptor |
| I - :PRO258 | 4.89413 | Hydrophobic | Pi-Alkyl | I | Pi-Orbitals | :PRO258 | Alkyl |
| I - :PRO258 | 3.93971 | Hydrophobic | Pi-Alkyl | I | Pi-Orbitals | :PRO258 | Alkyl |
| **Compound J. Polygonumnolide C2**  **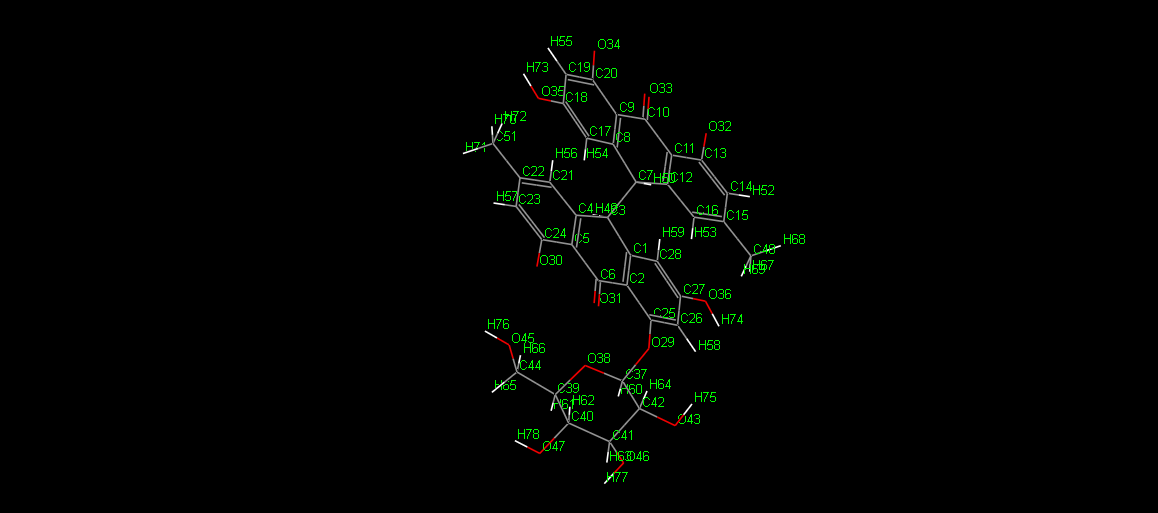** | | | | | | | |
| :ASN264:HD21 - J:O32 | 2.31179 | Hydrogen Bond | Conventional Hydrogen Bond | :ASN264:HD21 | H-Donor | J:O32 | H-Acceptor |
| J:H75 - J:O30 | 1.82831 | Hydrogen Bond | Conventional Hydrogen Bond | J:H75 | H-Donor | J:O30 | H-Acceptor |
| J:H64 - J:O31 | 2.52071 | Hydrogen Bond | Carbon Hydrogen Bond | J:H64 | H-Donor | J:O31 | H-Acceptor |
| J:O32 - :PHE457 | 4.35095 | Electrostatic | Pi-Anion | J:O32 | Negative | :PHE457 | Pi-Orbitals |
| :TRP454 - J | 5.63471 | Hydrophobic | Pi-Pi Stacked | :TRP454 | Pi-Orbitals | J | Pi-Orbitals |
| J:C48 - :PRO258 | 4.06993 | Hydrophobic | Alkyl | J:C48 | Alkyl | :PRO258 | Alkyl |
| J:C51 - :PRO258 | 5.34267 | Hydrophobic | Alkyl | J:C51 | Alkyl | :PRO258 | Alkyl |
| :TRP454 - J:C48 | 4.41415 | Hydrophobic | Pi-Alkyl | :TRP454 | Pi-Orbitals | J:C48 | Alkyl |
| J - :PRO258 | 4.82815 | Hydrophobic | Pi-Alkyl | J | Pi-Orbitals | :PRO258 | Alkyl |
| **Compound K. Polygonumnolide C3**  **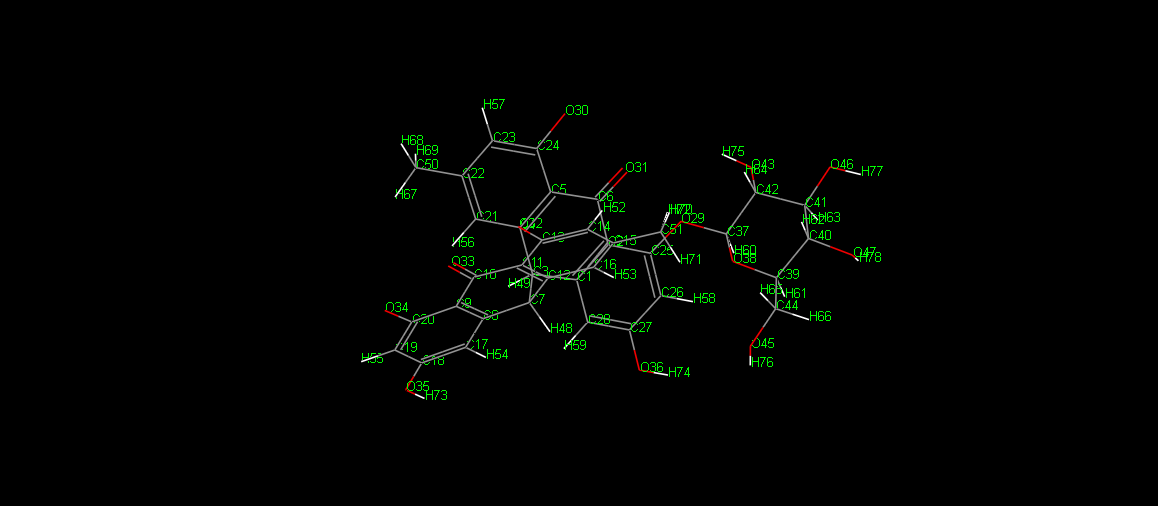** | | | | | | | |
| :ASN264:HD21 - K:O30 | 2.75487 | Hydrogen Bond | Conventional Hydrogen Bond | :ASN264:HD21 | H-Donor | K:O30 | H-Acceptor |
| K:H74 - :PRO258:O | 2.03406 | Hydrogen Bond | Conventional Hydrogen Bond | K:H74 | H-Donor | :PRO258:O | H-Acceptor |
| K:H76 - K:O31 | 2.08165 | Hydrogen Bond | Conventional Hydrogen Bond | K:H76 | H-Donor | K:O31 | H-Acceptor |
| :ASN264:HA - K:O30 | 2.61414 | Hydrogen Bond | Carbon Hydrogen Bond | :ASN264:HA | H-Donor | K:O30 | H-Acceptor |
| K:H64 - K:O31 | 2.60654 | Hydrogen Bond | Carbon Hydrogen Bond | K:H64 | H-Donor | K:O31 | H-Acceptor |
| K:C50 - :PRO258 | 4.4537 | Hydrophobic | Alkyl | K:C50 | Alkyl | :PRO258 | Alkyl |
| :TRP454 - K:C50 | 4.87602 | Hydrophobic | Pi-Alkyl | :TRP454 | Pi-Orbitals | K:C50 | Alkyl |
| :TRP454 - K:C50 | 3.65137 | Hydrophobic | Pi-Alkyl | :TRP454 | Pi-Orbitals | K:C50 | Alkyl |
| :TRP454 - K:C51 | 5.27102 | Hydrophobic | Pi-Alkyl | :TRP454 | Pi-Orbitals | K:C51 | Alkyl |
| K - :PRO258 | 4.64515 | Hydrophobic | Pi-Alkyl | K | Pi-Orbitals | :PRO258 | Alkyl |
| **Compound L. Polygonumnolide C4**  **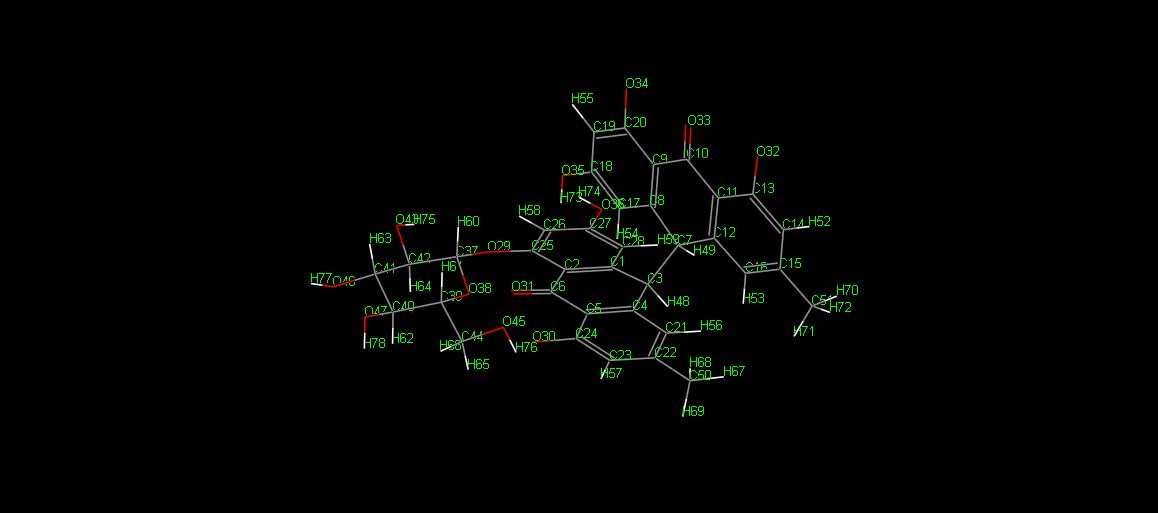** | | | | | | | |
| :ASN264:HD21 - L:O46 | 2.51057 | Hydrogen Bond | Conventional Hydrogen Bond | :ASN264:HD21 | H-Donor | L:O46 | H-Acceptor |
| :MET265:HN - L:O47 | 2.26416 | Hydrogen Bond | Conventional Hydrogen Bond | :MET265:HN | H-Donor | L:O47 | H-Acceptor |
| L:H75 - L:O31 | 2.39735 | Hydrogen Bond | Conventional Hydrogen Bond | L:H75 | H-Donor | L:O31 | H-Acceptor |
| L:H76 - L:O32 | 2.86441 | Hydrogen Bond | Conventional Hydrogen Bond | L:H76 | H-Donor | L:O32 | H-Acceptor |
| L:H76 - L:O33 | 2.01092 | Hydrogen Bond | Conventional Hydrogen Bond | L:H76 | H-Donor | L:O33 | H-Acceptor |
| L:H77 - :PRO263:O | 2.08378 | Hydrogen Bond | Conventional Hydrogen Bond | L:H77 | H-Donor | :PRO263:O | H-Acceptor |
| L:H60 - L:O31 | 2.57246 | Hydrogen Bond | Carbon Hydrogen Bond | L:H60 | H-Donor | L:O31 | H-Acceptor |
| L:H60 - L:O33 | 2.86367 | Hydrogen Bond | Carbon Hydrogen Bond | L:H60 | H-Donor | L:O33 | H-Acceptor |
| L:H61 - L:O33 | 2.54156 | Hydrogen Bond | Carbon Hydrogen Bond | L:H61 | H-Donor | L:O33 | H-Acceptor |
| L:H61 - L:O34 | 2.41148 | Hydrogen Bond | Carbon Hydrogen Bond | L:H61 | H-Donor | L:O34 | H-Acceptor |
| L:H62 - :PRO263:O | 2.45435 | Hydrogen Bond | Carbon Hydrogen Bond | L:H62 | H-Donor | :PRO263:O | H-Acceptor |
| L:H64 - :PRO263:O | 2.9928 | Hydrogen Bond | Carbon Hydrogen Bond | L:H64 | H-Donor | :PRO263:O | H-Acceptor |
| L:H66 - :MET265:O | 2.95517 | Hydrogen Bond | Carbon Hydrogen Bond | L:H66 | H-Donor | :MET265:O | H-Acceptor |
| L:O32 - L | 4.9309 | Electrostatic | Pi-Anion | L:O32 | Negative | L | Pi-Orbitals |
| L:O34 - :PHE457 | 4.97306 | Electrostatic | Pi-Anion | L:O34 | Negative | :PHE457 | Pi-Orbitals |
| L - L | 4.78601 | Hydrophobic | Pi-Pi Stacked | L | Pi-Orbitals | L | Pi-Orbitals |
| L - L | 4.48012 | Hydrophobic | Pi-Pi Stacked | L | Pi-Orbitals | L | Pi-Orbitals |
| **Compound M. Bilirubin**  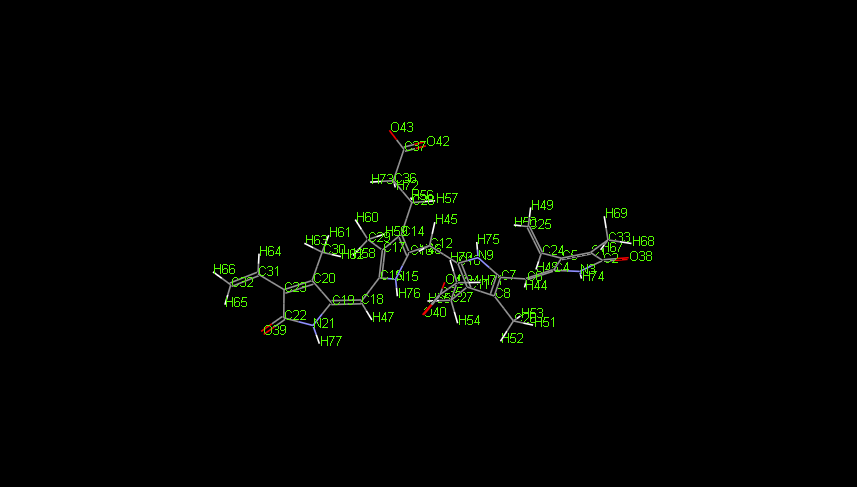 | | | | | | | |
| :PHE283:HA - M:O43 | 2.72591 | Hydrogen Bond | Carbon Hydrogen Bond | :PHE283:HA | H-Donor | M:O43 | H-Acceptor |
| :TYR286:HA - M:O38 | 2.77253 | Hydrogen Bond | Carbon Hydrogen Bond | :TYR286:HA | H-Donor | M:O38 | H-Acceptor |
| :ALA285 - M:C33 | 3.59472 | Hydrophobic | Alkyl | :ALA285 | Alkyl | M:C33 | Alkyl |
| M:C29 - :ILE343 | 4.54607 | Hydrophobic | Alkyl | M:C29 | Alkyl | :ILE343 | Alkyl |
| M:C29 - :VAL345 | 4.06591 | Hydrophobic | Alkyl | M:C29 | Alkyl | :VAL345 | Alkyl |
| M:C32 - :LYS346 | 4.63157 | Hydrophobic | Alkyl | M:C32 | Alkyl | :LYS346 | Alkyl |
| M - :ILE343 | 5.2329 | Hydrophobic | Pi-Alkyl | M | Pi-Orbitals | :ILE343 | Alkyl |
| M - :VAL345 | 4.85493 | Hydrophobic | Pi-Alkyl | M | Pi-Orbitals | :VAL345 | Alkyl |

**Table S2.** The parameters of the active sites of UGT1A1.
